# Efficacy of an All-Natural Polyherbal Mouthwash in Patients With Periodontitis: A Single-Blind Randomized Controlled Trial

**DOI:** 10.3389/fphys.2019.00632

**Published:** 2019-05-22

**Authors:** Scilla Sparabombe, Riccardo Monterubbianesi, Vincenzo Tosco, Giulia Orilisi, Andrell Hosein, Luigi Ferrante, Angelo Putignano, Giovanna Orsini

**Affiliations:** ^1^Department of Clinical Sciences and Stomatology, Marche Polytechnic University, Ancona, Italy; ^2^Department of Clinical and Molecular Sciences, Marche Polytechnic University, Ancona, Italy; ^3^Department of Biomedical Sciences and Public Health, Marche Polytechnic University, Ancona, Italy

**Keywords:** mouthwash, all-natural polyherbal, periodontitis, full mouth bleeding score, full mouth plaque score, probing depth, clinical attachment level, randomized controlled trial

## Abstract

**Aim:** This study aimed to evaluate the anti-inflammatory effect and the incidence of adverse effects of an all-natural polyherbal mouthwash in patients with periodontitis, after 3 months of use. These aims were accomplished by using full mouth bleeding score (FMBS), full mouth plaque score (FMPS), probing depth (PD) clinical attachment level (CAL) and a questionnaire recording any adverse events.

**Methods:** The present randomized controlled clinical study considered 40 patients with moderate or severe periodontitis, randomized in two groups: a test group (TG) and a control group (CG). TG was instructed to use a polyherbal mouthwash composed of *Propolis* resin extract, *Plantago lanceolata, Salvia officinalis* leaves extract, and 1.75% of essential oils and the CG was given a placebo mouthwash. Both groups were instructed to rinse for 2 min, twice daily after their routine oral home care with the different mouthwashes. Clinical measurements of FMBS, FMPS, PD and CAL were recorded at baseline (T0) and after 3 months (T1). The incidence of adverse outcomes was recorded at every follow-up. Mann–Whitney U test and Wilcoxon signed-rank test were used for the statistical analysis (*p* < 0.05).

**Results:** The final study sample consisted of 34 healthy individuals, 17 individuals in each of the two groups. TG and CG showed a statistically significant reduction in FMBS (*p* = 0.001 TG; *p* = 0.002 CG), FMPS (*p* = 0.001 TG; *p* = 0.003 CG), PD (*p* = 0.001 TG; *p* = 0.011 CG) and CAL (*p* < 0.001 TG; *p* = 0.020 CG) values from baseline to 3 months. The TG showed a statistically significant decrease in FMBS and FMPS compared with the CG. No adverse events or side effects were reported or observed in both groups.

**Conclusion:** The use of polyherbal mouthwash in patients with moderate or severe periodontitis has proved safe and effective in reducing bleeding score and plaque accumulation, after 3 months, compared with placebo, although no difference between the two groups were reported on PD and CAL (both improving at T1).

## Introduction

Mechanical plaque control has an essential role in the prevention of periodontal disease, however, it is not sufficiently effective alone (Page and Eke, 2007; Sälzer et al., 2015; Tonetti et al., 2015). A meta-analysis provided strong evidence in favor of the use of antimicrobial agents as adjuncts to mechanical plaque control (Gunsolley, 2006). The author highlighted two types of mouthwashes with consistent antiplaque and anti-gingivitis effects: chlorhexidine gluconate and mouthwash containing essential oils, such as menthol (0.042%), thymol (0.064%), methyl salicylate (0.060%) and eucalyptol (0.092%), which are commercially available. Despite the potent bactericidal action of chlorhexidine, there have been many reported side effects, mainly when used for long periods, such as taste alteration, supragingival calculus formation, extrinsic tooth staining and desquamation of oral mucosa (Lang and Brecx, 1986; Eick et al., 2011). Furthermore, chlorhexidine has been shown to induce cytotoxic and genotoxic effects in cells (Liu et al., 2018).

The increasingly widespread use of the mouthwashes for long periods has led research into the direction of finding effective and safe products, with a greater focus on herbal drugs. Plant extracts can be used as an alternative to chlorhexidine digluconate as their polyphenols compounds exhibit antimicrobial effects. A recent systematic review and meta-analysis, out of a total of 9 articles, investigated the efficacy of daily rinsing with a green tea-based herbal mouthwash in terms of plaque index (PI) and/or gingival index (Mathur et al., 2018). They demonstrated that the herbal mouthwash was not significantly different compared to the standard chlorhexidine-based mouthwashes in reducing plaque and gingival inflammation. Three main groups of plant polyphenols (stilbenes, flavonoids, and proanthocyanidins) were found to exhibit activity against caries, periodontitis and candidiasis in pre-clinical studies, however, there was a lack of strong evidence, regarding randomized clinical trials (Varoni et al., 2012).

Another recently published meta-analyses and meta-regression selected 16 studies comparing a notorious mouthwash containing plant-derived essential oils to placebo solution, cetylpyridinium chloride (CPC) and flossing in the proximal area (Haas et al., 2016). The authors concluded that, in patients with gingivitis, a notorious mouthwash was more efficacious for the reduction of plaque and gingival inflammation than mechanical plaque control either alone or in combination with CPC mouthwash. Due to the proven effectiveness of plant extracts against periodontal disease, there are many commercially available mouthwashes which include one or more active ingredients derived from plants. However, most of them also include additional contents that are artificial and chemically synthesized in the laboratory, being therefore not all-natural (Springhouse, 2003). Indeed, some studies report that they are not entirely innocuous (Hammer and Heel, 2012; Azzimonti et al., 2015).

A recently made commercially available mouthwash includes, in addition to essential oils, a combination of other natural products such as *Propolis* resin extract, *Plantago lanceolata, Salvia officinalis* leaves extract. It contains no artificial or chemically synthesized ingredients and is therefore entirely natural. There exists no randomized controlled trial that supports the safety and effectiveness of its specific formulation.

The aim of this study was to evaluate the safety and anti-inflammatory effect of the latter all-natural, commercially available polyherbal mouthwash in patients with periodontitis, by comparing it with a placebo mouthwash, after 3 months of use. This was accomplished by measuring the differences, between the two groups, in the following clinical outcomes: full mouth bleeding score (FMBS), full mouth plaque score (FMPS), probing depth (PD) level ≥ 5 mm and clinical attachment level (CAL) ≥ 4 mm (O’Leary, 1967; Ainamo and Bay, 1975). The incidence of adverse outcomes (the probability of the occurrence of side effects must be the same in the two groups) was also recorded using a questionnaire.

## Materials and Methods

Forty (40) volunteers, age range 20–65 years, were recruited for the current single-blind randomized placebo-controlled clinical trial. This study was performed in the Outpatient Department of Clinical Sciences and Stomatology of the Polytechnic University of Marche, Ancona, (Italy) between February and June 2017. This study was carried out in accordance with the recommendations of the Ethics Committee of the Azienda Ospedaliero-Universitaria Ospedali Riuniti, Ancona (Protocol N. 2017-0087 UN) and was registered in the Australian New Zealand Clinical Trials Registry (number of trial: ACTRN12618001192279). All subjects gave written informed consent in accordance with the Declaration of Helsinki.

### Study Population

After taking a detailed medical history and an initial clinical and radiologic examination, healthy individuals with a minimum of 20 teeth, smoker and no smoker, were selected. Clinical parameters for inclusion were: diagnosis of severe (at least 2 interproximal sites with CAL ≥ 6 mm and 1 interproximal site with PD ≥ 5 mm) or moderate (at least 2 interproximal sites with CAL ≥ 4 mm or 2 interproximal sites with PD ≥ 5 mm) periodontitis according to the Page and Eke classification (Page and Eke, 2007).

Exclusion criteria included: the use of antibiotics and anti-inflammatory drugs in previous 6 months; individuals with orthodontic or prosthetic appliances that could interfere with evaluation; individuals with an allergy to any ingredients used in the study; pregnant or lactating females; motor skills disorder.

All eligible volunteers were given oral and written information about the products and the purpose of the study and were asked to sign an informed consent.

### Study Design

This clinical study was designed as a randomized, 3-month placebo-controlled, single-blind clinical trial. A single examiner (SS), with more than 8 years of practice, collected the following clinical parameters: bleeding index, using FMBS and PI, using FMPS, on 6 surfaces; PD obtained by counting the number of pockets equal or greater than 5 mm; CAL recording the values equal to or greater than 4 mm, on 6 surfaces.

**FIGURE 1:**
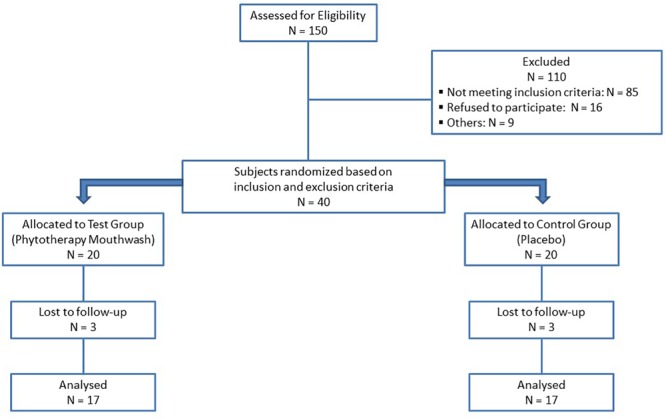
CONSORT flow chart. N: Number of subjects.

The subjects were randomly divided into two groups, through a computer-generated random table (Orsini et al., 2013). A CONSORT-type diagram explaining the design of this study is presented in Figure 1. At baseline (T0), after the clinical parameters assessment, all the participants received a thorough scaling and polishing to remove all plaque, stains and calculus, using ultrasonic scalers and hand instruments. For 3 months, a polyherbal mouthwash was prescribed to test group (TG) and a placebo mouthwash to the control group (CG). The polyherbal mouthwash (Pural Colluttorio, Fitomedical snc, Binasco, Milan, Italy) containing *Propolis* resin extract (1:3), *Plantago lanceolata* leaves extract (1:10), *Salvia officinalis* leaves extract (1:1) and 1.75% of essential oils from *Salvia officinalis, Syzygium aromaticum* buds, *Mentha piperita* leaves, *Commiphora myrrha* oleoresin and *Pistacia lentiscus* oleoresin, was made indistinguishable by the label’s absence in 100 ml opaque brown bottles marked only with the patient’s number. The placebo mouthwash was prepared with the following ingredients: 2 ml of glycerin (sweetening agent), cinnamon and vanilla flavoring agents, and brown food coloring, dissolved in 1 liter of distilled water and placed in 100 ml opaque brown bottles marked only with patient’s number.

All participants were instructed to perform their routine oral home care (brushing, flossing or interproximal cleaning) twice daily and, immediately after, to rinse with 1 ml mouthwash diluted in a measuring cup (15 ml) of water for 2 min. All patients were instructed to not rinse/eat anything for 30 min after mouthwash use.

Participants were followed up every 20 days and were asked to bring the empty bottles of the tested mouthwash. Each participant was asked to complete a written questionnaire to assess for any adverse effects related to use of mouthwash at day 20, 40, 60, 80 and 3 months, such as a change in taste sensations, soreness and redness of oral mucosa/tongue/gingiva, feeling of dryness, burning or discoloration. After 3 months (T1), the FMBS, FMPS, PD and CAL parameters were re-collected.

### Statistical Analyses

The differences of the single clinical parameters (FMBS, FMPS, PD level ≥ 5 mm, CAL ≥ 4 mm) were evaluated (Cosyn et al., 2013). For the same clinical score, the difference between TG and CG was carried out using the Mann–Whitney U test both at T0 and T1 (*p* < 0.05). Within each group, the differences in all clinical scores between T0 and T1 were evaluated using the Wilcoxon signed-rank test (*p* < 0.05). The difference in the rate of adverse events between the two groups were evaluated using the Fisher exact test for each time period.

## Results

Forty subjects were randomized based on the inclusion and exclusion criteria. There were six dropouts: one due to health reasons, four due to personal reasons and one excluded because subsequently submitted to a drug therapy. In total, 34 subjects completed the trial (17 each group, see Figure 1). Table 1 presents demographic data of the study population. There were no significant differences in mean age, sex and smoke of individuals between the two groups. At T0, there were no statistical differences in FMBS between the CG and TG; both groups showed a significant reduction in FMBS from T0 to T1. At T1, the TG group showed a statistically significant decrease in FMBS (*p* = 0.002) compared with the CG group (Table 2).

**Table 1:** Basic demographic characteristics of study population.

Parameter	Test (*n* = 17)	Control (*n* = 17)
Mean age ± SD (years)	51,05 ± 7,7	51,29 ± 10,2
Age range (years)	30 to 62	30 to 62
Males/females	8/9	8/9
Smokers/no smokers	6/11	6/11

**Table 2:** Values (mean ± SD) for Plaque and bleeding index and PD and CAL at Baseline and at 3 months.

Parameter	Interval	Test	Control	*P value*
FMBS	T0	12.23 ± 9.49	9.19 ± 6.23	*0.438*
	T1	1.63 ± 1.74	3.85 ± 2.68	*0.002*
	*P value*	*<0.001*	*0.002*	
FMPS	T0	44.70 ± 6.36	44.21 ± 16.04	*0.138*
	T1	17.64 ± 5.59	29.70 ± 14.38	*0.005*
	*P value*	*<0.001*	*0.003*	
PD ≥ 5 mm	T0	8.23 ± 14,42	2.36 ± 3.21	*0.068*
	T1	1.12 ± 1.79	1.37 ± 2.90	*0.820*
	*P value*	*0.001*	*0.011*	
CAL ≥ 4 mm	T0	43.25 ± 19.65	33.40 ± 16.28	*0.174*
	T1	29.17 ± 17.66	27.30 ± 17.39	*0.708*
	*P value*	*<0.001*	*0.020*	

*Statistically significant at *P* < 0.05. FMBS, full mouth bleeding score; FMPS, full mouth plaque score; PD, probing depth; CAL, clinical attachment level.*

At T0, the two groups showed no significant differences in mean of FMPS values, full-mouth PD and CAL. At T1, the TG group showed a significant decrease in FMPS (*p* = 0.005) compared with the CG group. Moreover, there were no significant differences in mean of full-mouth PD and CAL between the two groups at T1: both groups showed a significant reduction in PD (*p* = 0.001 TG; *p* = 0.011 CG) and CAL (*p* < 0.001 TG; *p* = 0.020 CG) values from baseline to 3 months (Table 2). There were no subjective complaints, adverse outcomes or side effects reported or observed in both groups at day 20, 40, 60, 80 and 3 months (Table 3).

**Table 3:** Side effects evaluation at each time period (day 20,40,60,80, 3 months) in the test (using polyherbal all-natural mouthwash) and Placebo groups.

SIDE effects		Test group (*n* = 17)	Control group (*n* = 17)
Soft tissue	Redness oral mucosa/tongue/gingiva	0	0
	Soreness oral mucosa/tongue/gingiva	0	0
	Mouth burning	0	0
	Desquamation	0	0
	Oral dryness	0	0
Hard tissue	Brown spots	0	0
Other	Taste perturbation (loss of taste)	0	0

## Discussion

The effectiveness of natural products and plant extracts is evidenced by several studies on its use in the treatment of oral diseases (Palombo, 2011; Škrovánková et al., 2012; Chinsembu, 2016). We conducted the first-ever randomized, controlled trial to investigate the effectiveness of a new combination of completely natural plant extracts (*Propolis* resin extract, *Plantago lanceolata, Salvia officinalis* leaves extract, and 1.75% of essential oils) in a single mouthwash available on the market, by comparing it with a placebo mouthwash.

The effectiveness of other herbal mouthwashes compared to a placebo mouthwash in patients with gingival or periodontal disease has been shown. Despite this, the topic is still controversial, due to some studies not using standardized methodology or having very short follow-ups. The effectiveness of three herbal extracts (*Juniperus communis, Urtica dioica, Achillea millefolium* on plaque and gingivitis) was studied by comparing it to a placebo mouthwash for 3 months, finding no beneficial effects (Weijden et al., 1998). Another study which investigated bacterial enumeration, plaque accumulation and gingival bleeding of a commercial herbal mouthwash containing *Salvadora persica* extract versus placebo over 3 weeks, found a statistically significant decrease in the PI of test subjects; a significant reduction in gingival bleeding was observed in both test and placebo groups (Khalessi et al., 2004).

Formulated in 2001, based on the traditional and historical uses of its functional ingredients and their components, the tested polyherbal mouthwash is all-natural. Numerous studies have highlighted essential oils antimicrobial effects even against multi-resistant bacteria (Mayaud et al., 2008; Chandra Shekar et al., 2015; Chinsembu, 2016). Because of their complex chemical composition, which includes more than 100 different therapeutic compounds, essential oils have a broad biological and antimicrobial activity spectrum: antibacterial, antifungal, and antiviral (Burt, 2004). *Plantago* species have considerable antiviral, anti-inflammatory, and antioxidant activities (Gálvez et al., 2005). *Plantago lanceolata* phenolic compounds seem to play a potential role in the control of bacterial growth and pathogenic oral flora virulence (Smullen et al., 2007). The antimicrobial effect of *sage* extract has been shown experimentally and clinically (Bozin et al., 2007; Geuenich et al., 2008). *Pistacia lentiscus* is an evergreen plant of the *Anacardiaceae* family, commonly found in the Mediterranean region; its fruits, galls, resin, and leaves have a long tradition in folk medicine. Recently, *Pistacia lentiscus* was found to exhibit an anti-inflammatory activity by reduction of inflammatory mediator production and inhibition of leukocyte recruitment to the inflammatory site (Ben Khedir et al., 2016). It also exhibited an antioxidant effect as a source of antioxidant molecules and indirectly as a stimulator of the activity and the expression of an antioxidant enzyme (Ben Khedir et al., 2016). *Propolis* is a well-known resinous material collected by bees; more than 300 components have been identified in *Propolis*, revealing that its composition is dependent upon the plant source and local flora. Several researchers have reported its antibacterial, antiviral, antitumor, anti-inflammatory property and immunomodulatory action (Sforcin, 2016; Oryan et al., 2018).

There is therefore strong beneficial evidence for these individual plant extracts in the tested mouthwash; however, no evidence exists for the combination of these plant extracts, formulated as a polyherbal mouthwash. This may be due to the quality of the substances and its delicate production process. Essential oils are unstable and fragile volatile compounds; they could be easily degraded by either oxidation, volatilization, heat or light if they are not protected from external factors, especially during the collection, storage and processing phase (Asbahani et al., 2015). *In vivo* studies and clinical trials on other herbal mouthwash have been performed (Khalessi et al., 2004; Haffajee et al., 2006), however, the need for clinical trials about the safety and efficacy of the combination of these herbal extracts in the tested mouth has been highlighted (Pan et al., 2013; Freires and Rosalen, 2016).

In the present study, the results are very encouraging; in both groups there was a significant reduction of inflammation from T0 to T1, confirming what is well described in the literature: mechanical therapy (scaling and polishing) improves clinical conditions by lowering the microbial load either by physical removal of plaque and by radical alteration of the subgingival habitat (Khalessi et al., 2004; Haffajee et al., 2006). However, in this study, 90 days after mechanical therapy, the TG had a greater reduction also in inflammation (FMBS) compared to the CG (*p* = 0.002).

A recent study investigated whether the adjunctive use of a mouthwash containing three natural essential oils (*Cymbopogon flexuosus* oil, *Thymus Zygis* oil, *Rosmarinus officinalis*) following sub-gingival debridement and scaling and root planning (SRP) could improve the clinical results regarding changes of the clinical parameters of PD, CAL and bleeding on probing (BoP) (Azad et al., 2016). Significant improvements of AL, PD, and BoP occurred in both groups after 3 months (*p* < 0.001); PD and BoP being significantly lower in the TG. In accordance with Azad study, our trial shows a significant improvement of PD (*P* = 0.001) and CAL (*P* < 0.001) in both groups, which can be explained because each subject in both groups had a preliminary scale and polishing at T0. Contrarily, no difference in PD and CAL between the two groups was revealed. This finding may be explained by the fact that PD reduction and gain in CAL are the expected result of mechanical instrumentation alone (Claffey et al., 1988; Haffajee et al., 1997). Therefore, mouthwashes do not seem crucial for these latter clinical outcomes. In support of our findings, another 3-month double-blind, randomized placebo-controlled trial studying clinical and microbial effects of an essential oil mouthwash in periodontal patients did not demonstrate any significant differences in full-mouth PD and full-mouth CAL between the test and placebo group (Cosyn et al., 2013). Indeed, plaque and bleeding indexes showed a significant reduction over time (*P* = 0.029); but, contrary to our data, there were no statically significant differences between the two groups (Cosyn et al., 2013).

The present single blind randomized controlled study shows a significant PI reduction in the TG and CG group. The considerable improvement of plaque control in both groups could be attributed to the Hawthorne effect; the subjects modified their oral hygiene routine due to the awareness of being observed (McCarney et al., 2007). However, FMPS has a significant difference between the two groups (*P* = 0.005), being significantly decreased in the TG. Our results are in agreement with other studies which investigated the anti-plaque and antigingivitis effects of a commercially available mouthwash containing essential oils with proven efficacy in individuals with and without periodontal disease (Sharma et al., 2004; Cortelli et al., 2009). However, although some herbal mouthwash showed side effects, mainly a mild mouth burning sensation (Manipal et al., 2016), the tested combination of all-natural polyherbal mouthwash has not demonstrated any discomfort or adverse events (Table 3). This study highlights the superior efficacy of the tested polyherbal mouthwash, over an essential oil mouthwash, as an agent without side effects, having considerable properties in controlling oral inflammation and microbiota. At this regard, a very recent comparative *in vitro* study demonstrated that chlorhexidine-based mouthwashes are still the most effective in regulating microbial homeostasis (Ardizzoni et al., 2018). However, chlorhexidine has several side effects that must be pondered when prescribing mouthwashes containing this molecule. Indeed, to the best of our knowledge, the need to develop alternative, innocuous but effective solutions are highly motivated. Therefore, despite the limitation that the polyherbal mouthwash was not compared to the “gold standard” chlorhexidine mouthwash and was only compared to a placebo, its all-natural ingredients have to be kept in great attention, since the preliminary evidence of effectiveness and safety is very promising.

## Conclusion

Common oral diseases such as gingivitis and periodontitis are based on microorganisms; herbal mouthwashes have an integral role in controlling the oral microbiota, and therefore are a useful adjunct in preventing periodontal disease. The use of the polyherbal mouthwash for 3 months significantly reduces inflammation (FMBS) and plaque accumulation (FMPS), showing a beneficial effect in patients with moderate or severe periodontitis. The most important finding is that, in bleeding and plaque score, participants using the all-natural polyherbal mouthwash showed significant improvement compared with the control. Noteworthy is that no adverse events or side effects were reported or observed in both groups at each time period. Further studies, in the near future, will be planned to compare it with the chlorhexidine mouthwash or other natural mouthwashes with proven efficacy.

## Data Availability

The datasets for this manuscript are not publicly available because the dataset is not publicly available as it is the property of the Polytechnic University of Marche prior to its publication. Requests to access the datasets should be directed to the corresponding author.

## Ethics Statement

This study was carried out in accordance with the recommendations of the Ethics Committee of the Azienda Ospedaliero-Universitaria Ospedali Riuniti, Ancona (Protocol N. 2017-0087 UN) and was registered in the Australian New Zealand Clinical Trials Registry (number of trial: ACTRN12618001192279). All subjects gave written informed consent in accordance with the Declaration of Helsinki.

## Author Contributions

SS and RM contributed to collection of data, conception and design, data analysis and interpretation, manuscript writing, critical reading and editing of the manuscript, and final revision and approval of the manuscript. VT and GuO assembled the data, analyzed and interpreted the data, wrote the manuscript, and approved the final version of the manuscript. GoO and AH interpreted the data, wrote and edited the manuscript, and approved the final version of the manuscript. LF, AP, and GoO contributed to conception and design, statistical analysis and interpretation, manuscript writing, and final approval and revision of the manuscript.

## Conflict of Interest Statement

The authors declare that the research was conducted in the absence of any commercial or financial relationships that could be construed as a potential conflict of interest.
